# New Flavones, a 2-(2-Phenylethyl)-4*H*-chromen-4-one Derivative, and Anti-Inflammatory Constituents from the Stem Barks of *Aquilaria sinensis*

**DOI:** 10.3390/molecules201119736

**Published:** 2015-11-24

**Authors:** Sin-Ling Wang, Tsong-Long Hwang, Mei-Ing Chung, Ping-Jyun Sung, Chih-Wen Shu, Ming-Jen Cheng, Jih-Jung Chen

**Affiliations:** 1 School of Pharmacy, College of Pharmacy, Kaohsiung Medical University, Kaohsiung 80708, Taiwan; u102530006@cc.kmu.edu.tw (S.-L.W.); meinch@kmu.edu.tw (M.-I.C.); 2 Graduate Institute of Natural Products, College of Medicine, Chang Gung University, Taoyuan 33302, Taiwan; htl@mail.cgu.edu.tw; 3 Research Center for Industry of Human Ecology and Graduate Institute of Health Industry Technology, Chang Gung University of Science and Technology, Taoyuan 33302, Taiwan; 4 Immunology Consortium, Chang Gung Memorial Hospital, Taoyuan 33302, Taiwan; 5 National Museum of Marine Biology and Aquarium, Pingtung 94450, Taiwan; pjsung@nmmba.gov.tw; 6 Department of Medical Education and Research, Kaohsiung Veterans General Hospital, Kaohsiung 81362, Taiwan; cwshu@vghks.gov.tw; 7 Bioresource Collection and Research Center (BCRC), Food Industry Research and Development Institute (FIRDI), Hsinchu 30062, Taiwan; cmj@firdi.org.tw; 8 Department of Pharmacy, Tajen University, Pingtung 90741, Taiwan

**Keywords:** *Aquilaria sinensis*, Thymelaeaceae, structure elucidation, flavone, 2-(2-phenylethyl)-4*H*-chromen-4-one, anti-inflammatory activity

## Abstract

In the current study, two new flavones, 4′-*O*-geranyltricin (**1**) and 3′-*O*-geranylpolloin (**2**), and a new 2-(2-phenylethyl)-4*H*-chromen-4-one derivative, 7-hydroxyl-6-methoxy-2-(2-phenylethyl)chromone (**3**), have been isolated from the stem barks of *A. sinensis*, together with 21 known compounds **4**–**24**. The structures of new compounds **1**–**3** were determined through spectroscopic and MS analyses. Compounds **2**, **3**, **5**, **6**, and **8**–**10** exhibited inhibition (IC_50_ ≤ 12.51 μM) of superoxide anion generation by human neutrophils in response to formyl-l-methionyl-l-leucyl-l-phenylalanine/cytochalasin B (fMLP/CB). Compounds **3**, **6**, **8**, **10**, and **19** inhibited fMLP/CB-induced elastase release with IC_50_ values ≤ 15.25 μM. This investigation reveals bioactive isolates (especially **2**, **3**, **5**, **6**, **8**, **9**, **10**, and **19**) could be further developed as potential candidates for the treatment or prevention of various inflammatory diseases.

## 1. Introduction

*Aquilaria sinensis* (Lour.) Gilg. (Thymelaeaceae) is an evergreen tree, distributed in southern China [1]. *A.* *sinensis*, locally called “Chen Xiang”, is used in China as a folk medicine for treatment of circulatory disorders, abdominal pain, vomiting, and dyspnea [1]. Its leaf has been commercially used as a functional tea with anti-diabetes activity [2]. Benzenoids [3,4,5,6,7], flavonoids [8,9,10,11,12,13], 2-(2-phenyl-ethyl)-4*H*-chromen-4-ones [4,10,14,15,16,17,18,19], steroids [19,20,21,22], sesquiterpenoids [21,23], triterpenoids [4], and their derivatives were isolated from this plant in previous studies.

**Figure 1:**
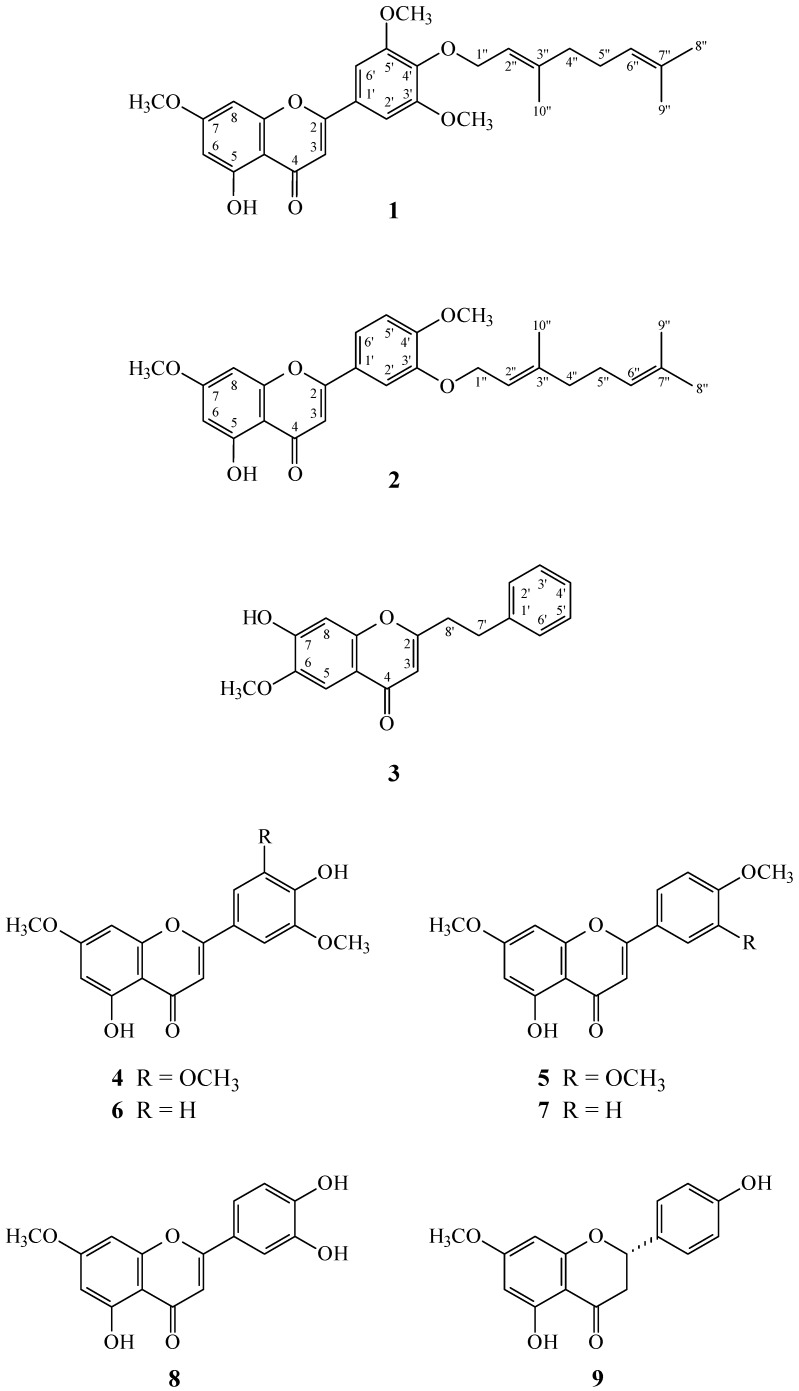

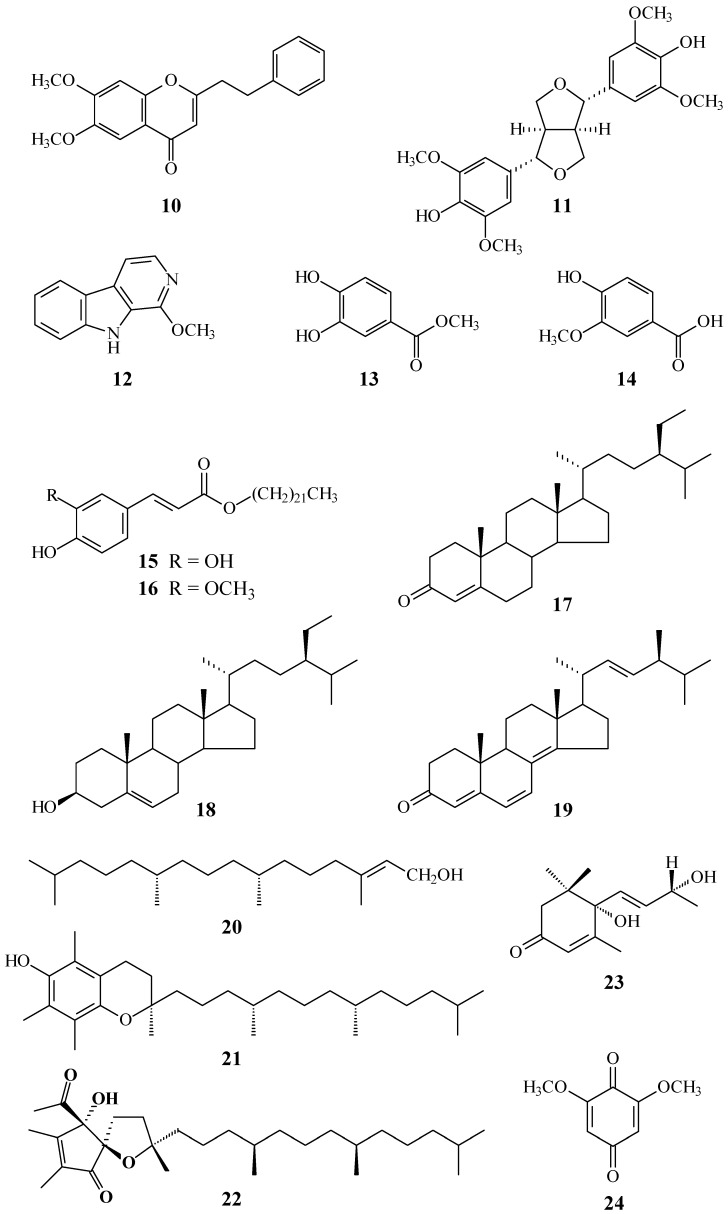
The chemical structures of new compounds **1**–**3** and known compounds **4**–**24** isolated from *A.* *sinensis*.

Many of these compounds were found to exhibit antitumor [5,18], nitrite scavenging [13], anti-acetylcholinesterase [23], and anti-inflammatory [12,19] activities. In our studies on the anti-inflammatory constituents of Formosan plants and Chinese herbal medicines, many species have been screened for *in vitro* inhibitory activity on neutrophil pro-inflammatory responses, and *A.* *sinensis* was found to be an active species. The MeOH extract of stem barks of *A.* *sinensis* showed potent inhibitory effects on superoxide anion generation and elastase release by human neutrophils in response to formyl-l-methionyl-l-leucyl-l-phenylalanine/cytochalasin B (fMLP/CB). Figure 1 illustrates the structures of two new flavones, 4′-*O*-geranyltricin (**1**) and 3′-*O*-geranylpolloin (**2**), and a new 2-(2-phenylethyl)-4*H*-chromen-4-one derivative, 7-hydroxyl-6-methoxy-2-(2-phenylethyl)-chromone (**3**) isolated from this extract. Twenty-one known compounds **4**–**24**, have also been isolated and identified from the stem barks of *A.* *sinensis* and their structures are depicted in Figure 1. This paper describes the structural elucidation of the compounds numbered **1** through **3**, and the inhibitory activities of all isolates on superoxide generation and elastase release by neutrophils.

## 2. Results

Chromatographic purification of the EtOAc-soluble fraction of a MeOH extract of stem barks of *A.* *sinensis* on a silica gel column and preparative thin-layer chromatography (TLC) afforded three new compounds **1**–**3** and twenty-one known compounds **4**–**24**.

**Figure 2:**
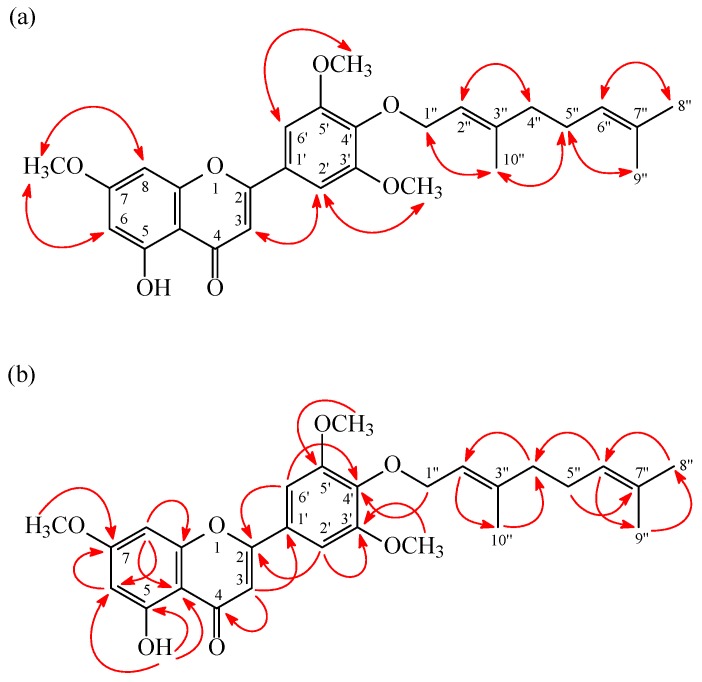
NOESY (**a**) and HMBC (**b**) correlations of **1**.

4′-*O*-Geranyltricin (**1**) was isolated as yellowish needles. Its molecular formula, C_28_H_32_O_7_, was determined on the basis of the positive HRESIMS peak at *m/z* 481.22185 [M + H]^+^ (calcd 481.22208) and this was supported by the ^1^H-, ^13^C-, and DEPT-NMR data. The IR spectrum showed the presence of OH (3408 cm^−1^) and carbonyl (1657 cm^−1^) groups. Comparison of the ^1^H-NMR data of **1** with those of tricin (**4**) [24,25] suggested that their structures were closely related, except that the 4′-geranyloxy group signals at δ 1.59 (3H, br s, H-9′′), 1.67 (3H, br s, H-8′′), 1.68 (3H, br s, H-10′′), 2.03 (2H, m, H-4′′), 2.08 (2H, m, H-5′′), 4.64 (2H, d, *J* = 7.2 Hz, H-1′′), 5.07 (1H, br t, *J* = 7.2 Hz, H-6′′), and 5.56 (1H, br t, *J* = 7.2 Hz, H-2′′) of **1** replaced the 4′-hydroxy group of **4**. This was supported by HMBC correlations observed between H-1′′ (δ_H_ 4.64) and C-4′ (δ_C_ 140.4), C-2′′ (δ_C_ 119.9), and C-3′′ (δ_C_ 142.1), and by NOESY correlations observed between H-1′′ (δ_H_ 4.64) and OMe-3′/5′ (δ_H_ 3.95), H-2′′ (δ_H_ 5.56), and H-10′′ (δ_H_ 1.68). Furthermore, the full assignment of ^1^H- and ^13^C-NMR resonances of **1** was confirmed by the ^1^H-^1^H COSY, NOESY (Figure 2), DEPT, HSQC, and HMBC (Figure 2) experiments. On the basis of the above data, the structure of **1** was elucidated as 4′-*O*-geranyltricin.

**Figure 3:**
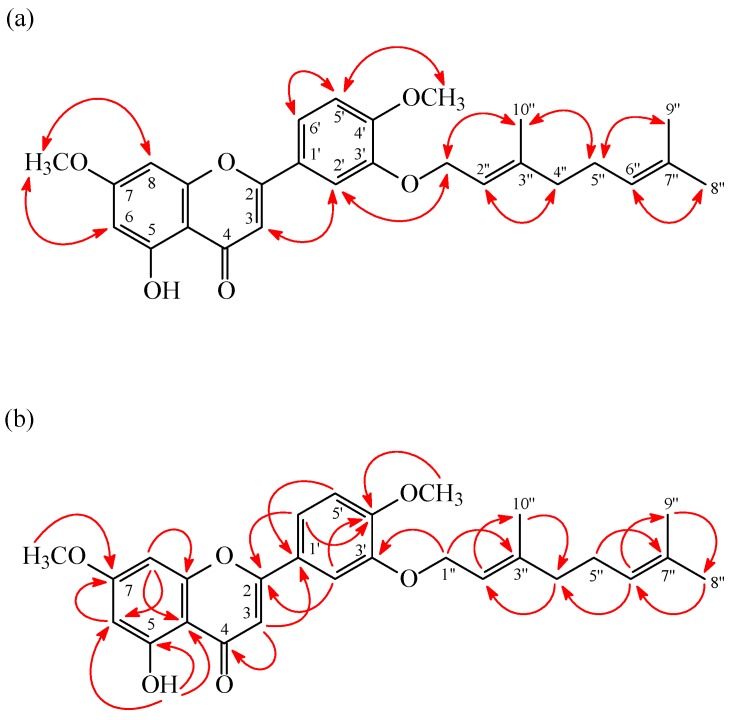
NOESY (**a**) and HMBC (**b**) correlations of **2**.

3′-*O*-Geranylpolloin (**2**) was obtained as yellowish needles. The molecular formula, C_27_H_30_O_6_, was deduced from a proton adduct ion at *m/z* 451.21133 [M + H]^+^ (calcd 451.21152) in the HRESI mass spectrum. IR absorptions for OH (3412 cm^−1^) and carbonyl (1660 cm^−1^) functions were observed. The ^1^H-NMR data of **2** were similar to 5-hydroxy-7,3′,4′-trimethoxyflavone (**5**) [26], except that the 3′-geranyloxy group [δ 1.58 (3H, br s, H-9′′), 1.64 (3H, br s, H-8′′), 1.81 (3H, br s, H-10′′), 2.10 (2H, m, H-4′′), 2.12 (2H, m, H-5′′), 4.72 (2H, d, *J* = 6.6 Hz, H-1′′), 5.07 (1H, br t, *J* = 6.6 Hz, H-6′′), 5.53 (1H, br t, *J* = 6.6 Hz, H-2′′)] of **2** replaced the OMe-3′ [δ3.99 (3H, s)] of **5**. This was supported by HMBC correlation observed between H-1′′ (δ_H_ 4.72) and C-3′ (δ_C_ 148.5), C-2′′ (δ_C_ 119.2), and C-3′′ (δ_C_ 141.6), and by NOESY correlations observed between H-1′′ (δ_H_ 4.72) and H-2′ (δ_H_ 7.36), H-2′′ (δ_H_ 5.53), and H-10′′ (δ_H_ 1.81). On the basis of the above data, the structure of **2** was elucidated as 3′-*O*-geranylpolloin, which was further confirmed by ^1^H-^1^H COSY and NOESY (Figure 3) experiments. The assignment of ^13^C-NMR resonances was confirmed by DEPT, HSQC and HMBC (Figure 3) techniques.

**Figure 4:**
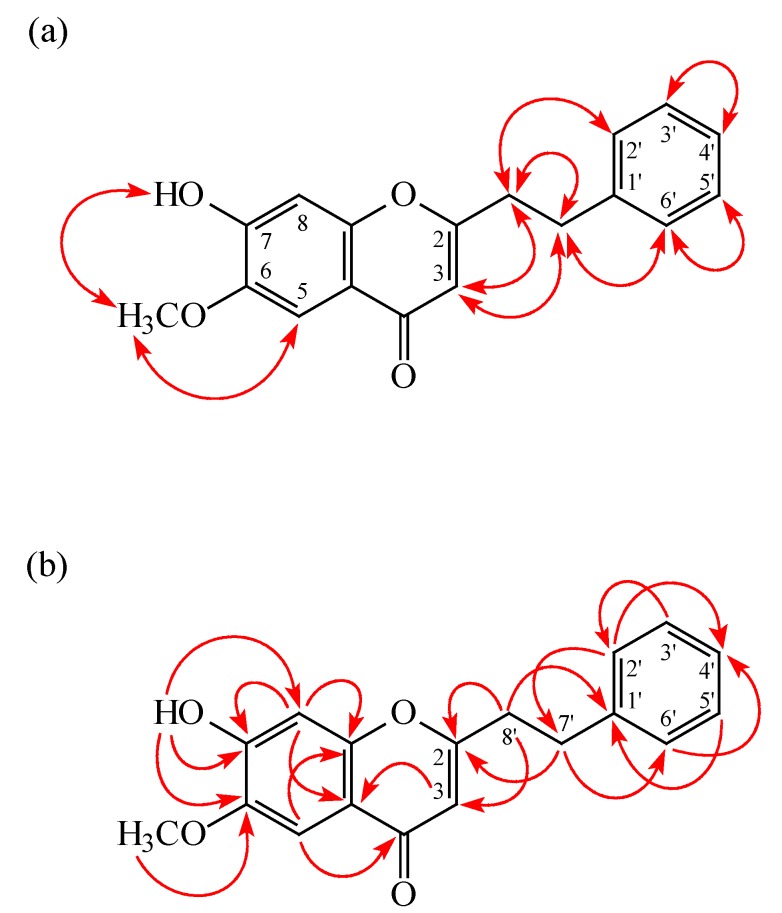
NOESY (**a**) and HMBC (**b**) correlations of **3**.

7-Hydroxy-6-methoxy-2-(2-phenylethyl)chromone (**3**) was obtained as a colorless prisms. The molecular formula C_18_H_16_O_4_ was deduced from a proton adduct ion peak at *m/z* 297.11209 [M + H]^+^ (calcd 297.11214) in the HRESI mass spectrum. The presence of hydroxy and carbonyl groups was revealed by the bands at 3418 and 1634 cm^−1^, respectively, in the IR spectrum. The ^1^H-NMR spectrum indicated the presence of a 2-phenylethyl group [δ 2.91 (2H, t, *J* = 7.5 Hz, H-8′), 3.04 (2H, t, *J* = 7.5 Hz, H-7′), 7.20 (2H, br d, *J* = 7.5 Hz, H-2′ and H-6′), 7.21 (1H, br t, *J* = 7.5 Hz, H-4′), 7.29 (2H, br t, *J* = 7.5 Hz, H-3′ and H-5′)], a methoxy group [δ 4.00 (3H, s, OMe-6)], a hydroxy group [δ 6.24 (1H, s, D_2_O exchangeable, OH-7)], and three singlet aromatic protons [δ 6.08 (1H, s, H-3), 6.94 (1H, s, H-8), 7.54 (1H, s, H-5)]. Comparison of the ^1^H and ^13^C-NMR data of **3** with those of 6,7-dimethoxy-2-(2-phenylethyl)chromone (**10**) [27] suggested that their structures were closely related, except that 7-hydroxy group [δ 6.24 (1H, s, D_2_O exchangeable, OH-7)] of **3** replaced the OMe-7 [δ 3.97 (3H, s)] of **10**. This was supported by HMBC correlations between OH-7 (δ 6.24) and C-6 (δ 145.1), C-7 (δ 151.2), and C-8 (δ 102.7) and NOESY correlations between OH-7 (δ 6.24) and OMe-6 (δ 4.00). Furthermore, the full assignment of ^1^H- and ^13^C-NMR resonances was confirmed by the ^1^H-^1^H COSY, NOESY (Figure 4), DEPT, HSQC, and HMBC (Figure 4) experiments. According to the above data, the structure of **3** was elucidated as 7-hydroxy-6-methoxy-2-(2-phenylethyl)chromone.

The known isolates were readily identified by a comparison of their physical and spectroscopic data (UV, IR, ^1^H-NMR, [α]_D_, and MS) with the corresponding authentic samples or literature values, and included six flavones: tricin (**4**) [25], 5-hydroxy-7,3′,4′-trimethoxyflavone (**5**) [26], velutin (**6**) [28], apigenin 7,4′-dimethyl ether (**7**) [29], 3′-hydroxygenkwanin (**8**) [30], and sakuranetin (**9**) [31], a 2-(2-phenylethyl)-4*H*-chromen-4-one, 6,7-dimethoxy-2-(2-phenylethyl)chromone (**10**) [27], a lignan, (–)-syringaresinol (**11**) [32], a β-carboline, taraxacine A (**12**) [33], four benzenoids, methyl 3,4-dihydroxybenzoate (**13**) [34], vanillic acid (**14**) [35], docosyl caffeate (**15**) [36], and docosyl *trans*-ferulate (**16**) [37], three steroids, β-sitostenone (**17**) [38], β-sitosterol (**18**) [39], and ergosta-4,6,8(14),22-tetraen-3-one (**19**) [40], a diterpene, *trans*-phytol (**20**) [41], two α-tocopheroids, α-tocopherol (**21**) [42] and α-tocospiro A (**22**) [43], a cyclohex-2-en-1-one, blumenol A (**23**) [44], and a benzoquinone, 2,6-dimethoxy-*p*-benzoquinone (**24**) [45].

Human neutrophils are known to play an important role in the host defense against microorganisms and in the pathogenesis of various diseases such as rheumatoid arthritis, ischemia-reperfusion injury, asthma, and chronic obstructive pulmonary disease [46,47]. In response to different stimuli, activated neutrophils secrete a series of cytotoxins, such as superoxide anion (O_2_^•−^), a precursor of other reactive oxygen species (ROS), granule proteases, bioactive lipids [46,48,49]. Suppression of the extensive or inappropriate activation of neutrophils by drugs has been proposed as a way to ameliorate inflammatory diseases. Reactive oxygen species (ROS) [e.g., superoxide anion (O_2_^•−^) and hydrogen peroxide] and granule proteases (e.g., elastase, cathepsin G, and proteinase-3) produced by human neutrophils are involved in the pathogenesis of a variety of inflammatory diseases. The effects on neutrophil pro-inflammatory responses of compounds isolated from the stem barks of *A. sinensis* were evaluated by suppressing fMet-Leu-Phe/cytochalasin B (fMLP/CB)-induced superoxide anion (O_2_^•−^) generation and elastase release by human neutrophils. The inhibitory activity data on neutrophil pro-inflammatory responses are summarized in Table 1.

**Table 1:** Inhibitory effects of compounds **1**–**24** from the stem barks of *A. sinensis* on superoxide radical anion generation and elastase release by human neutrophils in response to fMet-Leu-Phe/cytochalasin B.

Compound	IC_50_ (μM) ^a^ or (Inh %) ^b^	
	Superoxide Anion Generation	Elastase Release
4′-*O*-Geranyltricin (**1**)	(13.23 ± 6.82)	(12.80 ± 6.84)
3′-*O*-Geranylpolloin (**2**)	12.51 ± 2.75 ^e^	(17.34 ± 3.81) ^c^
7-Hydroxy-6-methoxy-2-(2-phenylethyl)chromone (**3**)	4.62 ± 1.48 ^e^	3.91 ± 0.87 ^e^
Tricin (**4**)	(3.61 ± 2.29)	(17.69 ± 1.71) ^e^
5-Hydroxy-7,3′,4′-trimethoxyflavone (**5**)	4.69 ± 0.94 ^e^	(9.32 ± 1.37) ^e^
Velutin (**6**)	1.78 ± 0.35 ^e^	4.26 ± 0.12 ^d^
Apigenin 7,4′-dimethyl ether (**7**)	elicit superoxide anion generation and elastase release	
3′-Hydroxygenkwanin (**8**)	7.96 ± 0.76 ^e^	4.56 ± 0.63 ^e^
Sakuranetin (**9**)	1.74 ± 0.17 ^e^	(23.84 ± 4.91) ^d^
6,7-Dimethoxy-2-(2-phenylethyl)chromone (**10**)	11.54 ± 2.19 ^e^	10.48 ± 1.35 ^d^
(–)-Syringaresinol (**11**)	(30.23 ± 1.71) ^e^	(25.12 ± 6.22) ^d^
Taraxacine A (**12**)	(14.64 ± 2.95) ^d^	(44.43 ± 1.90) ^e^
Methyl 3,4-dihydroxybenzoate (**13**)	(1.32 ± 2.25)	(19.13 ± 5.85) ^c^
Vanillic acid (**14**)	29.34 ± 6.01 ^e^	29.92 ± 2.50 ^e^
Docosyl caffeate (**15**)	(1.23 ± 5.08)	(25.57 ± 5.00) ^d^
Docosyl *trans*-ferulate (**16**)	(27.83 ± 4.37)	(27.12 ± 6.23)
β-Sitostenone (**17**)	(2.74 ± 0.96) ^c^	(3.92 ± 2.22)
β-Sitosterol (**18**)	(9.08 ± 6.13)	(2.43 ± 2.95)
Ergosta-4,6,8(14),22-tetraen-3-one (**19**)	(42.63 ± 1.82) ^d^	15.25 ± 3.75 ^c^
*trans*-Phytol (**20**)	(4.91 ± 5.52)	(22.65 ± 5.66) ^d^
α-Tocopherol (**21**)	(0.55 ± 2.51)	(9.37 ± 4.92)
α-Tocospiro A (**22**)	(0.55 ± 2.51)	(9.37 ± 4.92)
Blumenol A (**23**)	(1.37 ± 1.38)	(10.72 ± 1.62) ^d^
2,6-Dimethoxy-*p*-benzoquinone (**24**)	(47.09 ± 2.85) ^e^	(16.00 ± 5.53) ^c^
Diphenyleneiodonium	1.73 ± 0.72 ^e^	–
Phenylmethylsulfonyl fluoride	–	199.6 ± 30.7 ^e^

Diphenyleneiodonium and phenylmethylsulfonyl fluoride were used as positive control. Results are presented as averages ± SEM (*n* = 4). ^a^ Concentration necessary for 50% inhibition (IC_50_); ^b^ Percentage of inhibition (Inh %) at 30 μM; ^c^ *p* < 0.05 compared with the control; ^d^ *p* < 0.01 compared with the control; ^e^ *p* < 0.001 compared with the control.

Diphenyleneiodonium and phenylmethylsulfonyl fluoride were used as positive controls for O_2_^•−^ generation and elastase release, respectively. From the results of our biological tests, the following conclusions can be drawn: (a) 3′-*O*-Geranylpolloin (**2**), 7-hydroxy-6-methoxy-2-(2-phenylethyl)chromone (**3**), 5-hydroxy-7,3′,4′-trimethoxyflavone (**5**), velutin (**6**), 3′-hydroxy-genkwanin (**8**), sakuranetin (**9**), and 6,7-dimethoxy-2-(2-phenylethyl)chromone (**10**) exhibited potent inhibition (IC_50_ ≤ 12.51 μM) of superoxide anion (O_2_^•−^) generation by human neutrophils in response to fMLP/CB; (b) 7-hydroxy-6-methoxy-2-(2-phenylethyl)chromone (**3**), velutin (**6**), sakuranetin (**9**), 6,7-dimethoxy-2-(2-phenylethyl)chromone (**10**), and ergosta-4,6,8(14)-22-tetraen-3-one (**19**) exhibited potent inhibition (IC_50_ ≤ 15.25 μM) against fMLP-induced elastase release; (c) 7-hydroxy-6-methoxy-2-(2-phenylethyl)chromone (**3**) (with a 7-hydroxy group) exhibited more effective inhibition than its analogue, 6,7-dimethoxy-2-(2-phenylethyl)chromone (**10**) (with a 7-methoxy substituent) against fMLP-induced O_2_^•−^ generation and elastase release; (d) among the flavone analogues (**1**, **2**, and **4**–**8**), velutin (**6**) (with a 4′-hydroxy-3′-methoxyphenyl moiety) and 5-hydroxy-7,3′,4′-trimethoxyflavone (**5**) (with a 3′,4′-dimethoxyphenyl moiety) exhibited more effective inhibition than their analogues, **1**, **2**, **4**, **7**, and **8**, against fMLP-induced O_2_^•−^ generation; velutin (**6**) and 3′-hydroxygenkwanin (**8**) (with a 3′,4′-dihydroxyphenyl moiety) exhibited more effective inhibition than their analogues, **1**, **2**, **4**, **5**, and **7**, against fMLP-induced elastase release; (e) among the flavone analogues (**1**, **2**, and **4**–**8**), only apigenin 7,4′-dimethyl ether (**7**) (with a 4′-methoxyphenyl moiety) at 10 μg/mL alone elicited superoxide anion generation and elastase release by human neutrophils in the absence of fMLP/CB; (f) sakuranetin (**9**) and velutin (**6**) were the most effective among the isolated compounds, with IC_50_ values of 1.74 ± 0.17 and 1.78 ± 0.35 μM, against fMLP-induced superoxide anion generation; (g) 7-hydroxy-6-methoxy-2-(2-phenylethyl)chromone (**3**), velutin (**6**), and 3′-hydroxygenkwanin (**8**) were the most effective among the isolated compounds, with IC_50_ value of 3.91 ± 0.87, 4.26 ± 0.12, and 4.56 ± 0.63 μM, against fMLP-induced elastase release.

## 3. Discussion

Twenty four compounds, including two new flavones, 4′-*O*-geranyltricin (**1**) and 3′-*O*-geranylpolloin (**2**), and a new 2-(2-phenylethyl)-4*H*-chromen-4-one derivative, 7-hydroxy-6-methoxy-2-(2-phenylethyl)chromone (**3**) were isolated from the stem barks of *A. sinensis*. Known compounds **9**, **12**–**16**, and **19**–**24** were obtained from this plant for the first time. The structures of these compounds were established on the basis of spectroscopic data. Discovery of the two new flavones and a new 2-(2-phenylethyl)-4*H*-chromen-4-one derivative from the genus *Aquilaria* may not only provide more structure-activity data of flavones and 2-(2-phenylethyl)-4*H*-chromen-4-one, but may also contribute to enhancing our understanding of the taxonomy and evolution of the genus *Aquilaria*.

Granule proteases (e.g., elastase, cathepsin G) and reactive oxygen species (ROS) e.g., superoxide anion (O_2_^•−^), hydrogen peroxide] and produced by human neutrophils contribute to the pathogenesis of inflammatory diseases. Inhibition of the inappropriate activation of neutrophils by drugs has been proposed as a way to ameliorate inflammatory diseases. Based on the results of our biological tests (Table 1), 3′-*O*-geranylpolloin (**2**), 7-hydroxy-6-methoxy-2-(2-phenylethyl)chromone (**3**), 5-hydroxy-7,3′,4′-trimethoxyflavone (**5**), velutin (**6**), 3′-hydroxygenkwanin (**8**), sakuranetin (**9**), and 6,7-dimethoxy-2-(2-phenylethyl)chromone (**10**) were the most effective among these compounds, with IC_50_ values of 12.51 ± 2.75, 4.62 ± 1.48, 4.69 ± 0.94, 1.78 ± 0.35, 7.96 ± 0.76, 1.74 ± 0.17, and 11.54 ± 2.19 μM, respectively, against fMLP-induced superoxide anion generation. 7-Hydroxy-6-methoxy-2-(2-phenylethyl)chromone (**3**), velutin (**6**), 3′-hydroxygenkwanin (**8**), 6,7-di-methoxy-2-(2-phenylethyl)chromone (**10**), and ergosta-4,6,8(14),22-tetraen-3-one (**19**) exhibited the most effective among the isolates, with IC_50_ values of 3.91 ± 0.87, 4.26 ± 0.12, 4.56 ± 0.63, 10.48 ± 1.35, and 15.25 ± 3.75 μM, respectively, against fMLP-induced elastase release. Our study suggests *A. sinensis* and its isolates (especially **3**, **5**, **6**, and **8**–**10**) could be further developed as potential candidates for the treatment or prevention of various inflammatory diseases. More experiments should be performed to deduce the action modes of these compounds.

## 4. Experimental Section

### 4.1. Ethics Statement

Blood was taken from healthy human donors (20–30 years old) by venipuncture, using a protocol (No. 102-1595A3) approved by the Institutional Review Board at Chang Gung Memorial Hospital. All donors gave written consent. The Medical Ethics Committee of Chang Gung Memorial Hospital approved this consent procedure.

### 4.2. General Experimental Procedures

Melting points were determined on a Yanaco micro-melting point apparatus (Kyoto, Japan) and are uncorrected. Optical rotations were measured using a Jasco DIP-370 polarimeter (Jasco, Tokyo, Japan) in CHCl_3_. Ultraviolet (UV) spectra were obtained on a UV-240 spectrophotometer (Jasco). Infrared (IR) spectra (neat or KBr) were recorded on a 2000 FT-IR spectrometer (Perkin Elmer, Norwalk, CT, USA). The proton nuclear magnetic resonance (NMR) spectra were recorded on a Varian Unity Plus and Mercury 400 (Varian, Palo Alto, CA, USA), a Varian Inova 500 and a Varian Unity Plus 600 spectrometer operating at 400, 500 and 600 MHz, the carbon NMR spectra, including correlation spectroscopy (COSY), nuclear Overhauser effect spectrometry (NOESY), heteronuclear multiple-bond correlation (HMBC), heteronuclear single-quantum coherence (HSQC) experiments, were recorded on a Varian Unity Plus 600 spectrometer operating at 600 MHz (^1^H) and 150 MHz (^13^C), respectively, with chemical shifts given in ppm (δ) using tetramethylsilane (TMS) as an internal standard. Electrospray ionization (ESI), high-resolution electrospray ionization (HRESI) and electron ionization (EI)-mass spectra were recorded on an APEX II mass spectrometer (Bruker, Billerica, MA, USA) and Trace GC/Polaris Q MS (Thermo Finnigan, San Jose, CA, USA). Silica gel (70–230, 230–400 mesh) (Merck, Darmstadt, Germany) was used for column chromatography (CC). Silica gel 60 F-254 (Merck) was used for thin-layer chromatography (TLC) and preparative thin-layer chromatography (PTLC).

### 4.3. Plant Material

The stem barks of *A. sinensis* was collected from Pingtung County, Taiwan, in August 2013 and identified by Prof. J.J. Chen. A voucher specimen (AS 201308) was deposited in the Department of Pharmacy, Tajen University, Pingtung, Taiwan.

### 4.4. Extraction and Isolation

The dried stem barks (4.1 kg) of *A. sinensis* were pulverized and extracted three times with MeOH (20 L each) for 3 days. The MeOH extracts were concentrated under reduced pressure at 35 °C, and the residue (390 g) was partitioned between *n*-hexane and H_2_O (1:1). The *n*-hexane layer was concentrated to give a residue (fraction A, 93 g). The water layer was further extracted with EtOAc, and the EtOAc-soluble part (fraction B, 75 g) and the water-solubles (fraction C, 212 g) were separated. Fraction B (75 g) was chromatographed on silica gel (70–230 mesh, 3.2 kg), eluting with *n*-hexane, gradually increasing the polarity with acetone or MeOH to give 11 fractions: B1 (2 L, *n*-hexane/acetone, 20:1), B2 (2 L, *n*-hexane/acetone, 15:1), B3 (6.5 L, *n*-hexane/acetone, 10:1), B4 (5 L, *n*-hexane/acetone, 8:1), B5 (10.5 L, *n*-hexane/acetone, 5:1), B6 (2 L, *n*-hexane/acetone, 4:1), B7 (12 L, *n*-hexane/acetone, 3:1), B8 (5 L, *n*-hexane/acetone, 2:1), B9 (4 L, *n*-hexane/acetone, 1:1), B10 (3 L, acetone), and B11 (1 L, MeOH). Fraction B1 (5.1 g) was chromatographed further on silica gel (230–400 mesh, 230 g) eluting with CH_2_Cl_2_/EtOAc (100:1–0:1) to give 10 fractions (each 1.2 L, B1-1–B1-10). Fraction B1-2 (138 mg) was purified further by preparative TLC (silica gel, *n*-hexane/EtOAc, 15:1) to obtain α-tocopherol (**21**) (5.8 mg) (*R_f_* = 0.45). Fraction B1-5 (167 mg) was purified further by preparative TLC (silica gel, *n*-hexane/CH_2_Cl_2_, 1:5) to obtain α-tocospiro A (**22**) (4.6 mg) (*R_f_* = 0.38). Fraction B1-6 (153 mg) was purified further by preparative TLC (silica gel, *n*-hexane/EtOAc, 4:1) to yield β-sitostenone (**17**) (8.6 mg) (*R_f_* = 0.76). Fraction B2 (5.7 g) was chromatographed further on silica gel (230–400 mesh, 260 g) eluting with CH_2_Cl_2_/EtOAc (80:1–0:1) to give 12 fractions (each 900 mL, B2-1–B2-12). Fraction B2-3 (405 mg) was purified by MPLC (silica column, CH_2_Cl_2_/EtOAc 60:1–0:1) to afford six subfractions (each 200 mL, B2-3-1–B2-3-6). Fraction B2-3-3 (38 mg) was purified by preparative TLC (silica gel, *n*-hexane/acetone, 8:1) to obtain *trans*-phytol (**20**) (3.9 mg) (*R_f_* = 0.29). Fraction B2-6 (195 mg) was purified further by preparative TLC (silica gel, *n*-hexane/EtOAc, 5:1) to give ergosta-4,6,8(14),22-tetraen-3-one (**19**) (6.3 mg) (*R_f_* = 0.52). Fraction B3 (6.6 g) was chromatographed on silica gel (230–400 mesh, 300 g) eluting with CH_2_Cl_2_/MeOH (70:1–0:1) to give 10 fractions (each 1 L, B3-1–B3-10). Fraction B3-1 (210 mg) was purified further by preparative TLC (silica gel, *n*-hexane/EtOAc, 3:1) to yield tetracosyl *trans*-ferulate (**16**) (6.3 mg) (*R_f_* = 0.52). Fraction B3-4 (220 mg) was purified further by preparative TLC (silica gel, CH_2_Cl_2_/EtOAc, 60:1) to afford β-sitosterol (**18**) (10.7 mg) (*R_f_* = 0.52). Fraction B5 (10.9 g) was chromatographed on silica gel (230–400 mesh, 495 g) eluting with CH_2_Cl_2_/MeOH (50:1–0:1) to give 12 fractions (each 1.5 L, B5-1–B5-12). Fraction B5-2 (203 mg) was purified further by preparative TLC (silica gel, CH_2_Cl_2_/MeOH, 50:1) to yield 5-hydroxy-7,3′,4′-trimethoxyflavone (**5**) (8.3 mg) (*R_f_* = 0.84). Fraction B5-3 (198 mg) was purified further by preparative TLC (silica gel, CH_2_Cl_2_/EtOAc, 30:1) to yield 3′-*O*-geranylpolloin (**2**) (4.7 mg) (*R_f_* = 0.83). Fraction B5-6 (216 mg) was purified further by preparative TLC (silica gel, *n*-hexane/acetone, 11:10) to afford sakuranetin (**9**) (5.2 mg) (*R_f_* = 0.71), 6,7-dimethoxy-2-(2-phenylethyl)chromone (**10**) (4.6 mg) (*R_f_* = 0.62), and taraxacine-A (**12**) (5.1 mg) (*R_f_* = 0.45). Fraction B5-7 (187 mg) was purified further by preparative TLC (silica gel, *n*-hexane/EtOAc, 1:1) to yield tetracosyl caffeate (**15**) (4.7 mg) (*R_f_* = 0.76). Fraction B6 (5.6 g) was chromatographed further on silica gel (230–400 mesh, 255 g) eluting with CH_2_Cl_2_/acetone (25:1–0:1) to give 8 fractions (each 1.3 L, B6-1–B6-8). Fraction B6-1 (205 mg) was purified further by preparative TLC (silica gel, *n*-hexane/acetone, 3:2) to obtain apigenin 7,4′-dimethyl ether (**7**) (8.7 mg) (*R_f_* = 0.67). Fraction B6-3 (169 mg) was purified further by preparative TLC (silica gel, CH_2_Cl_2_/EtOAc, 10:1) to obtain 2,6-dimethoxy-1,4-benzoquinone (**24**) (5.3 mg) (*R_f_* = 0.64). Fraction B7 (8.3 g) was chromatographed on silica gel (230–400 mesh, 380 g) eluting with CH_2_Cl_2_/MeOH (20:1–0:1) to give 11 fractions (each 1.2 L, B7-1–B7-11). Fraction B7-1 (177 mg) was purified further by preparative TLC (silica gel, CH_2_Cl_2_/acetone, 25:1) to obtain velutin (6) (10.7 mg) (*R_f_* = 0.57). Fraction B7-5 (215 mg) was purified further by preparative TLC (silica gel, CH_2_Cl_2_/EtOAc, 30:1) to yield 3′-*O*-geranylpolloin (**1**) (4.1 mg) (*R_f_* = 0.71). Fraction B7-7 (220 mg) was purified further by preparative TLC (silica gel, *n*-hexane/EtOAc, 1:1) to obtain tricin (**4**) (9.4 mg) (*R_f_* = 0.57). Fraction B7-9 (203 mg) was purified further by preparative TLC (silica gel, *n*-hexane/acetone, 1:1) to obtain 7-hydroxy-6-methoxy-2-(2-phenylethyl)chromone (**3**) (5.2 mg) (*R_f_* = 0.79). Fraction B8 (6.3 g) was chromatographed on silica gel (230–400 mesh, 285 g) eluting with CH_2_Cl_2_/MeOH (12:1–0:1) to give 10 fractions (each 1 L, B8-1–B8-10). Fraction B8-4 (188 mg) was purified further by preparative TLC (silica gel, CH_2_Cl_2_/acetone, 6:1) to yield methyl 3,4-dihydroxybenzoate (**13**) (5.4 mg) (*R*_f_ = 0.50). Fraction B8-5 (193 mg) was purified further by preparative TLC (silica gel, *n*-hexane/EtOAc, 1:5) to obtain blumenol (**23**) (4.9 mg) (*R_f_* = 0.69). Fraction B8-6 (213 mg) was purified further by preparative TLC (silica gel, CH_2_Cl_2_/acetone, 5:1) to yield 3′-hydroxygenkwanin (**8**) (6.2 mg) (*R_f_* = 0.40). Fraction B8-7 (183 mg) was purified further by preparative TLC (silica gel, *n*-hexane/acetone, 3:2) to obtain vanillic acid (**14**) (7.9 mg) (*R_f_* = 0.23). Fraction B9 (5.3 g) was chromatographed on silica gel (230–400 mesh, 240 g) eluting with CH_2_Cl_2_/MeOH (10:1–0:1) to give 9 fractions (each 900 mL, B9-1–B9-9). Fraction B9-4 (189 mg) was purified further by preparative TLC (silica gel, *n*-hexane/acetone, 20:1) to obtain (‒)-syringaresinol (**11**) (6.8 mg) (*R_f_* = 0.48).

*4′-O-Geranyltricin* (**1**). Yellowish needles (CH_2_Cl_2_-MeOH); m.p. 256–258 °C; UV (MeOH) λ_max_ (log ε) 207 (4.67), 268 (4.14), 331 (4.21) nm; IR (KBr) ν_max_ 3408 (OH), 1657 (C=O) cm^−1^; ^1^H-NMR (CDCl_3_, 600 MHz) δ 1.59 (3H, br s, H-9′′), 1.67 (3H, br s, H-8′′), 1.68 (3H, br s, H-10′′), 2.03 (2H, m, H-4′′), 2.08 (2H, m, H-5′′), 3.89 (3H, s, OMe-7), 3.95 (6H, s, OMe-3′ and OMe-5′), 4.64 (2H, d, *J* = 7.2 Hz, H-1′′), 5.07 (1H, br t, *J* = 7.2 Hz, H-6′′), 5.56 (1H, br t, *J* = 7.2 Hz, H-2′′), 6.39 (1H, d, *J* = 2.1 Hz, H-6), 6.50 (1H, d, *J* = 2.1 Hz, H-8), 6.60 (1H, s, H-3), 7.08 (2H, s, H-2′ and H-6′), 12.73 (1H, s, D_2_O exchangeable, OH-5); ^13^C-NMR (CDCl_3_, 150 MHz) δ 16.4 (C-10′′), 17.6 (C-9′′), 25.6 (C-8′′), 26.4 (C-5′′), 39.6 (C-4′′), 55.8 (OMe-7), 56.4 (OMe-3′ and OMe-5′), 69.6 (C-1′′), 92.7 (C-8), 98.2 (C-6), 103.8 (C-2′ and C-6′), 105.6 (C-3), 105.6 (C-10), 119.9 (C-2′′), 123.9 (C-6′′), 126.4 (C-1′), 131.7 (C-7′′), 140.4 (C-4′), 142.1 (C-3′′), 154.1 (C-3′ and C-5′), 157.7 (C-9), 162.3 (C-5), 164.0 (C-2), 165.6 (C-7), 182.4 (C-4); ESIMS *m/z* 481 [M + H]^+^; HRESIMS *m/z* 481.22185 [M + H]^+^ (calcd for C_28_H_33_O_7_, 481.22208).

*3′-O-Geranylpolloin* (**2**). Yellowish needles (CH_2_Cl_2_-MeOH); m.p. 246–248 °C; UV (MeOH) λ_max_ (log ε) 205 (4.59), 250 (4.20), 268 (4.13), 339 (4.24) nm; IR (KBr) ν_max_ 3412 (OH), 1660 (C=O) cm^−1^; ^1^H-NMR (CDCl_3_, 600 MHz) δ 1.58 (3H, s, H-9′′), 1.64 (3H, s, H-8′′), 1.81 (3H, s, H-10′′), 2.10 (2H, m, H-4′′), 2.12 (2H, m, H-5′′), 3.89 (3H, s, OMe-7), 3.95 (3H, s, OMe-4′), 4.72 (2H, d, *J* = 6.6 Hz, H-1′′), 5.07 (1H, br t, *J* = 6.6 Hz, H-6′′), 5.53 (1H, br t, *J* = 6.6 Hz, H-2′′), 6.37 (1H, d, *J* = 2.4 Hz, H-6), 6.48 (1H, d, *J* = 2.4 Hz, H-8), 6.56 (1H, s, H-3), 6.97 (1H, d, *J* = 8.4 Hz, H-5′), 7.36 (1H, d, *J* = 2.4 Hz, H-2′), 7.51 (1H, dd, *J* = 8.4, 2.4 Hz, H-6′), 12.80 (1H, s, D_2_O exchangeable, OH-5); ^13^C-NMR (CDCl_3_, 150 MHz) δ 16.8 (C-10′′), 17.7 (C-9′′), 25.6 (C-8′′), 26.3 (C-5′′), 39.6 (C-4′′), 55.8 (OMe-7), 56.1 (OMe-4′), 66.2 (C-1′′), 92.7 (C-8), 98.0 (C-6), 104.6 (C-3), 105.6 (C-10), 111.0 (C-2′), 111.4 (C-5′), 119.2 (C-2′′), 120.1 (C-6′), 123.7 (C-1′), 123.7 (C-6′′), 131.9 (C-7′′), 141.6 (C-3′′), 152.9 (C-4′), 148.5 (C-3′), 157.7 (C-9), 164.1 (C-2), 165.5 (C-7), 182.4 (C-4); ESIMS *m/z* 451 [M + H]^+^; HRESIMS *m/z* 451.21133 [M + H]^+^ (calcd for C_27_H_31_O_6_, 451.21152).

*7-Hydroxy-6-methoxy-2-(2-phenylethyl)chromone* (**3**). Colorless prism (CH_2_Cl_2_); m.p. 181–183 °C; UV (MeOH) λ_max_ (log ε) 207 (4.69), 226 (4.56), 282 (4.11), 319 (4.15) nm; IR (KBr) ν_max_ 3418 (OH), 1634 (C=O) cm^−1^; ^1^H-NMR (CDCl_3_, 600 MHz) δ 2.91 (2H, t, *J* = 7.5 Hz, H-8′), 3.04 (2H, t, *J* = 7.5 Hz, H-7′), 4.00 (3H, s, OMe-6), 6.08 (1H, s, H-3), 6.24 (1H, s, D_2_O exchangeable, OH-7), 6.94 (1H, s, H-8), 7.20 (2H, br d, *J* = 7.5 Hz, H-2′ and H-6′), 7.21 (1H, br t, *J* = 7.5 Hz, H-4′),7.29 (2H, br t, *J* = 7.5 Hz, H-3′ and H-5′), 7.54 (1H, s, H-5) ; ^13^C-NMR (CDCl_3_, 150 MHz) δ 33.0 (C-7′), 36.0 (C-8′), 56.5 (OMe-6), 102.7 (C-8), 104.4 (C-5), 109.4 (C-3), 117.0 (C-10), 126.5 (C-4′), 128.3 (C-2′ and C-6′), 128.6 (C-3′ and C-5′), 139.8 (C-1′), 145.1 (C-6), 151.2 (C-7), 152.7 (C-9), 167.7 (C-2), 177.6 (C-4); ESIMS *m/z* 297 [M + H]^+^; HRESIMS *m/z* 297.11209 [M + H]^+^ (calcd for C_18_H_17_O_4_, 297.11214).

### 4.5. Biological Assay

The effect of the isolated compounds on neutrophil pro-inflammatory response was evaluated by monitoring the inhibition of superoxide anion generation and elastase release in fMLP/CB-activated human neutrophils in a concentration-dependent manner. The purity of the tested compounds was >98% as identified by NMR and MS.

#### 4.5.1. Preparation of Human Neutrophils

Human neutrophils from venous blood of healthy, adult volunteers (20–28 years old) were isolated using a standard method of dextran sedimentation prior to centrifugation in a Ficoll Hypaque gradient and hypotonic lysis of erythrocytes [50]. Purified neutrophils containing >98% viable cells, as determined by the trypan blue exclusion method [51], were re-suspended in a calcium (Ca^2+^)-free HBSS buffer at pH 7.4 and were maintained at 4 °C prior to use.

#### 4.5.2. Measurement of Superoxide Anion Generation

The assay for measurement of superoxide anion generation was based on the SOD-inhibitable reduction of ferricytochrome *c* [52,53]. In brief, after supplementation with 0.5 mg/mL ferricytochrome *c* and 1 mM Ca^2+^, neutrophils (6 × 10^5^/mL) were equilibrated at 37 °C for 2 min and incubated with different concentrations (10–0.01 μg/mL) of compounds or DMSO (as control) for 5 min. Cells were incubated with cytochalasin B (1 μg/mL) for 3 min prior to the activation with 100 nM formyl-l-methionyl-l-leucyl-l-phenylalanine for 10 min. Changes in absorbance with the reduction of ferricytochrome *c* at 550 nm were continuously monitored in a double-beam, six-cell positioner spectrophotometer with constant stirring (Hitachi U-3010, Tokyo, Japan). Calculations were based on differences in the reactions with and without SOD (100 U/mL) divided by the extinction coefficient for the reduction of ferricytochrome *c* (ε = 21.1/mM/10 mm).

#### 4.5.3. Measurement of Elastase Release

Degranulation of azurophilic granules was determined by measuring elastase release as described previously [53,54]. Experiments were performed using MeO-Suc-Ala-Ala-Pro-Val-*p*-nitroanilide as the elastase substrate. Briefly, after supplementation with MeO-Suc-Ala-Ala-Pro-Val-*p*-nitroanilide (100 μM), neutrophils (6 × 10^5^/mL) were equilibrated at 37 °C for 2 min and incubated with compounds for 5 min. Cells were stimulated with fMLP (100 nM)/CB (0.5 μg/mL), and changes in absorbance at 405 nm were monitored continuously in order to assay elastase release. The results were expressed as the percent of elastase release in the fMLP/CB-activated, drug-free control system.

#### 4.5.4. Statistical Analysis

Results are expressed as the mean ± SEM, and comparisons were made using Student’s *t*-test. A probability of 0.05 or less was considered significant. The software SigmaPlot was used for the statistical analysis.

## 5. Conclusions

Twenty four compounds, including two new flavones, 4′-*O*-geranyltricin (**1**) and 3′-*O*-geranylpolloin (**2**), and a new 2-(2-phenylethyl)-4*H*-chromen-4-one derivative, 7-hydroxyl-6-methoxy-2-(2-phenylethyl)chromone (**3**) were isolated from the stem barks of *A. sinensis*. The structures of these compounds were established on the basis of spectroscopic data. Reactive oxygen species (ROS) e.g., superoxide anion (O_2_^•−^), hydrogen peroxide] and granule proteases (e.g., elastase, cathepsin G) produced by human neutrophils contribute to the pathogenesis of inflammatory diseases. The effects on neutrophil pro-inflammatory responses of isolates were evaluated by suppressing fMLP/CB-induced O_2_^•−^ generation and elastase release by human neutrophils. The results of anti-inflammatory experiments indicate that compounds **2**, **3**, **5**, **6**, **8**–**10**, and **19** can significantly inhibit fMLP-induced O_2_^•−^ generation and/or elastase release. Sakuranetin (**9**) and 7-hydroxy-6-methoxy-2-(2-phenylethyl)chromone (**3**) were the most effective among the isolated compounds, with IC_50_ values of 1.74 ± 0.17 and 3.91 ± 0.87 μM, respectively, against fMLP-induced O_2_^•−^ generation and elastase release. Our study suggests *A. sinensis* and its isolates (especially **3**, **5**, **6**, and **8**–**10**) are worthy of further biomedical investigation and could be expectantly developed as potential candidates for the treatment or prevention of various inflammatory diseases.

## Supplementary Materials

ESI-MS, HR-ESI-MS, ^1^H-NMR, and ^13^C-NMR spectra of three new compounds **1**–**3** are available as Supplementary Information. Supplementary materials can be accessed at: http://www.mdpi.com/1420-3049/20/11/19736/s1.
