# Supplementary material for: New Flavones, a 2-(2-Phenylethyl)-4H-chromen-4-one Derivative, and Anti-Inflammatory Constituents from the Stem Barks of Aquilaria sinensis
Source: Molecules. 2015 Nov 24;20(11):20912–25. doi: 10.3390/molecules201119736 (PMC6332152; doi:10.3390/molecules201119736)
Supplement: Supplementary file 1 [file molecules-20-19736-s001.pdf]

Supplementary Materials: New Flavones, a 2-(2-Phenylethyl)-4*H*-chromen-4-one Derivative, and Anti-Inflammatory Constituents from the Stem Barks of *Aquilaria sinensis*

Sin-Ling Wang, Tsong-Long Hwang, Mei-Ing Chung, Ping-Jyun Sung, Chih-Wen Shu, Ming-Jen Cheng and Jih-Jung Chen

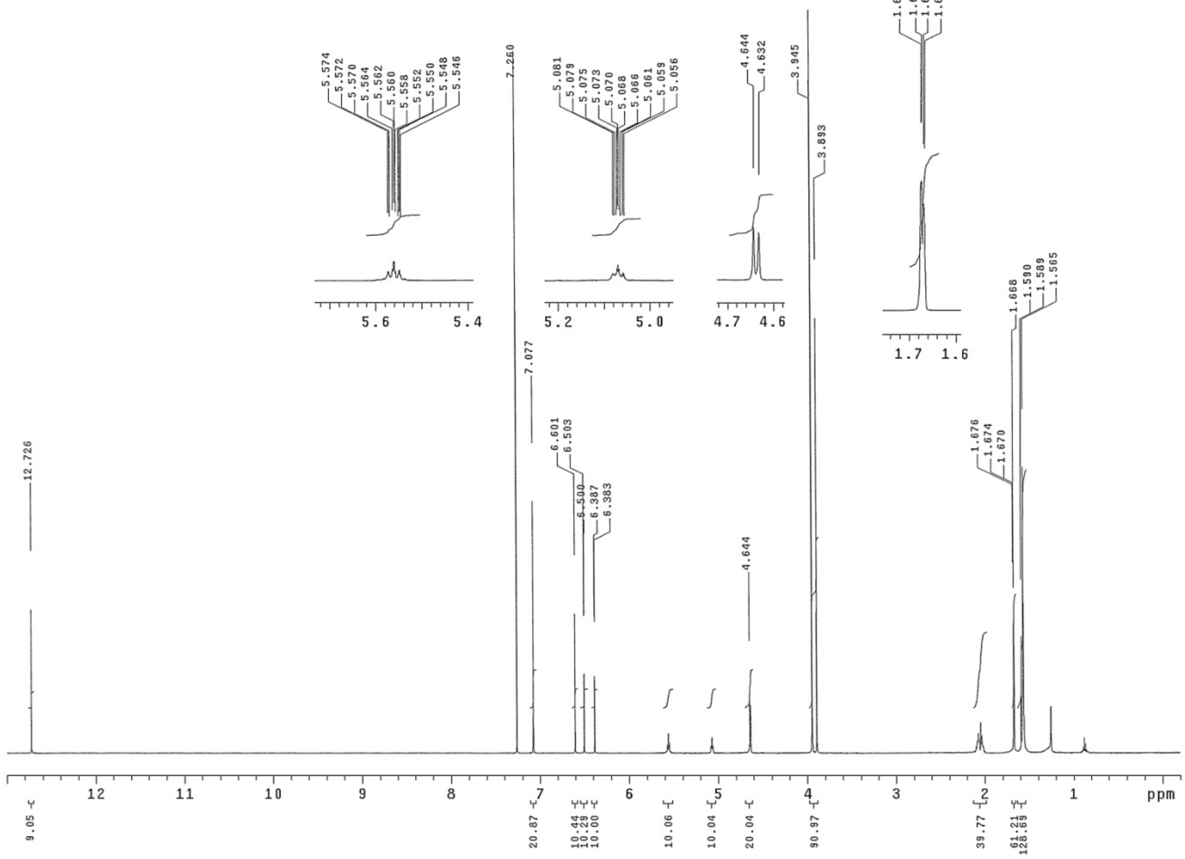

Figure S1. ^{1}H-NMR spectrum ($CDCl_3$, 600 MHz) of **1**.

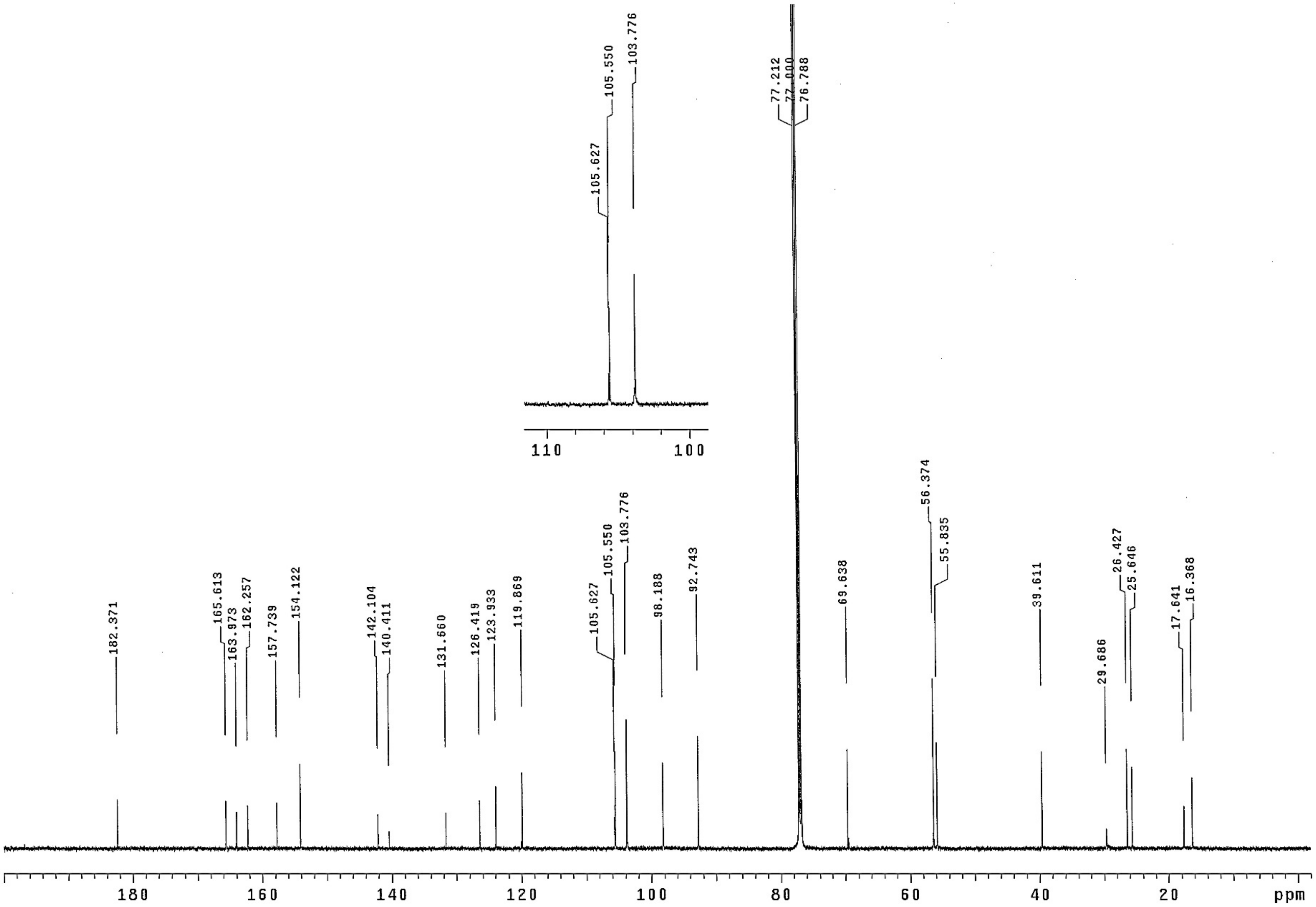

Figure S2. ^{13}C-NMR spectrum ($CDCl_3$, 150 MHz) of **1**.

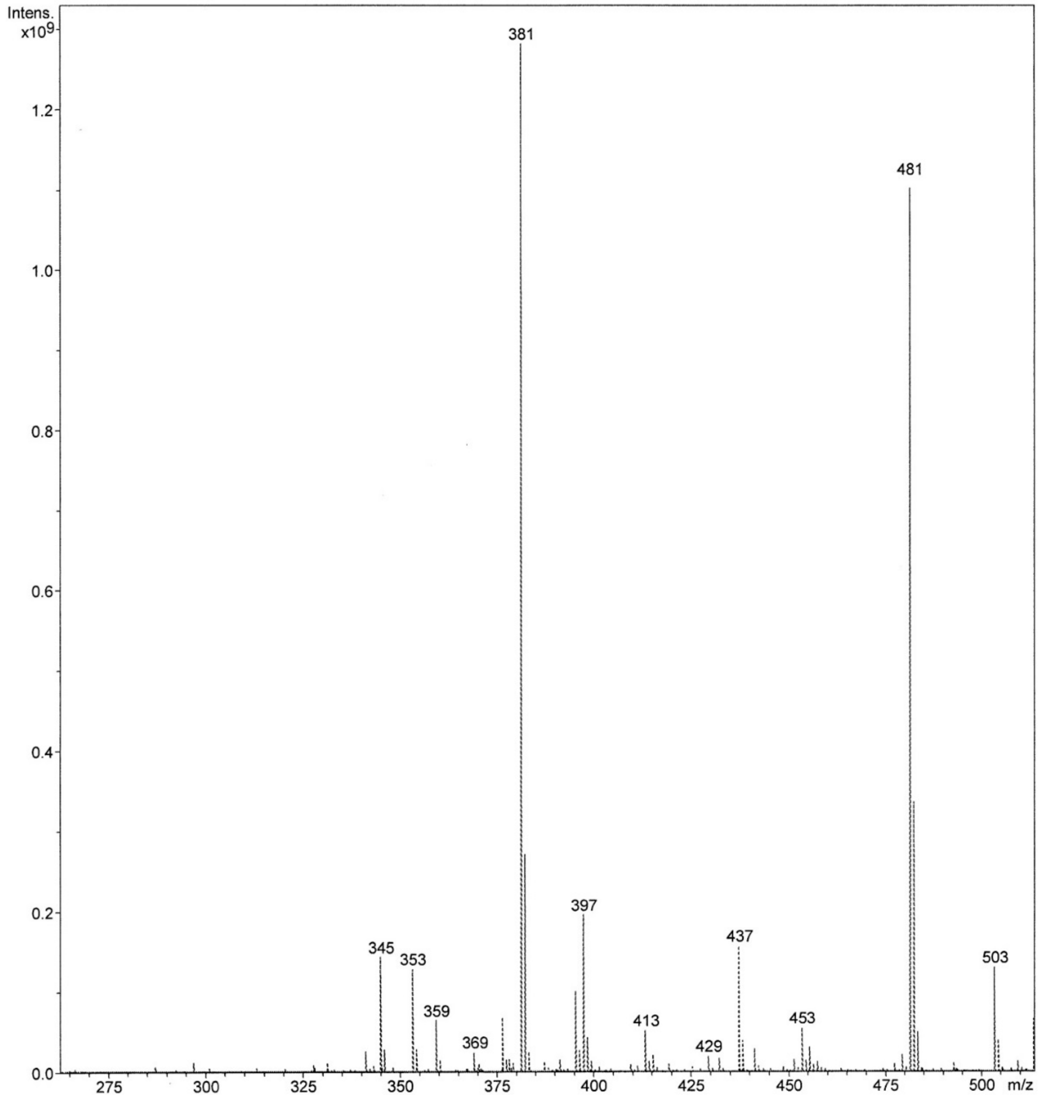

Figure S3. ESI-MS spectrum of **1**.

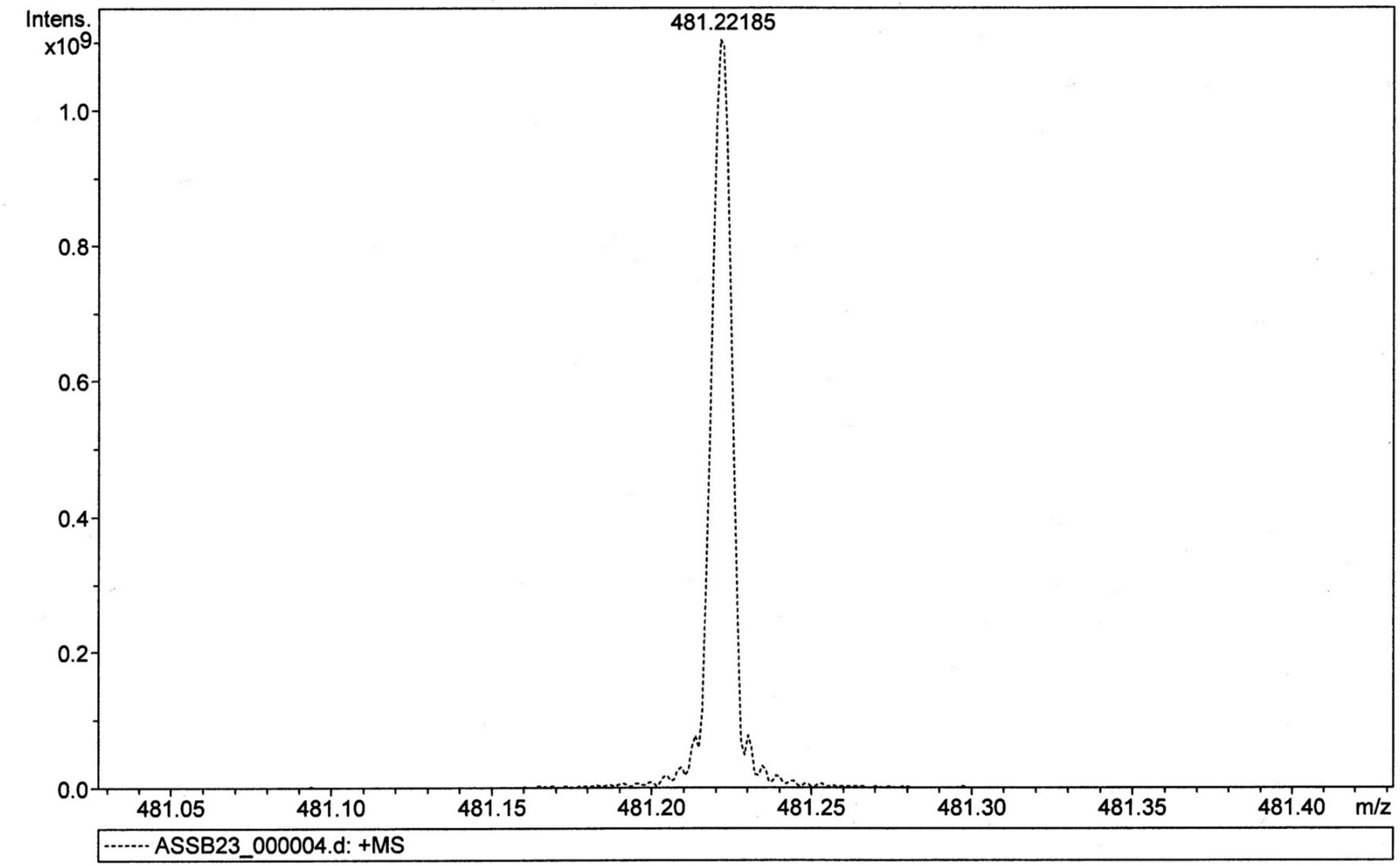

Meas. m/z	#	Formula	Score	m/z	err [mDa]	err [ppm]	mSigma	rdb	e^- Conf	N-Rule
481.22185	1	C 28 H 33 O 7	100.00	481.22208	0.23	0.47	7.7	12.5	even	ok

Figure S4. HR-ESI-MS spectrum of **1**.

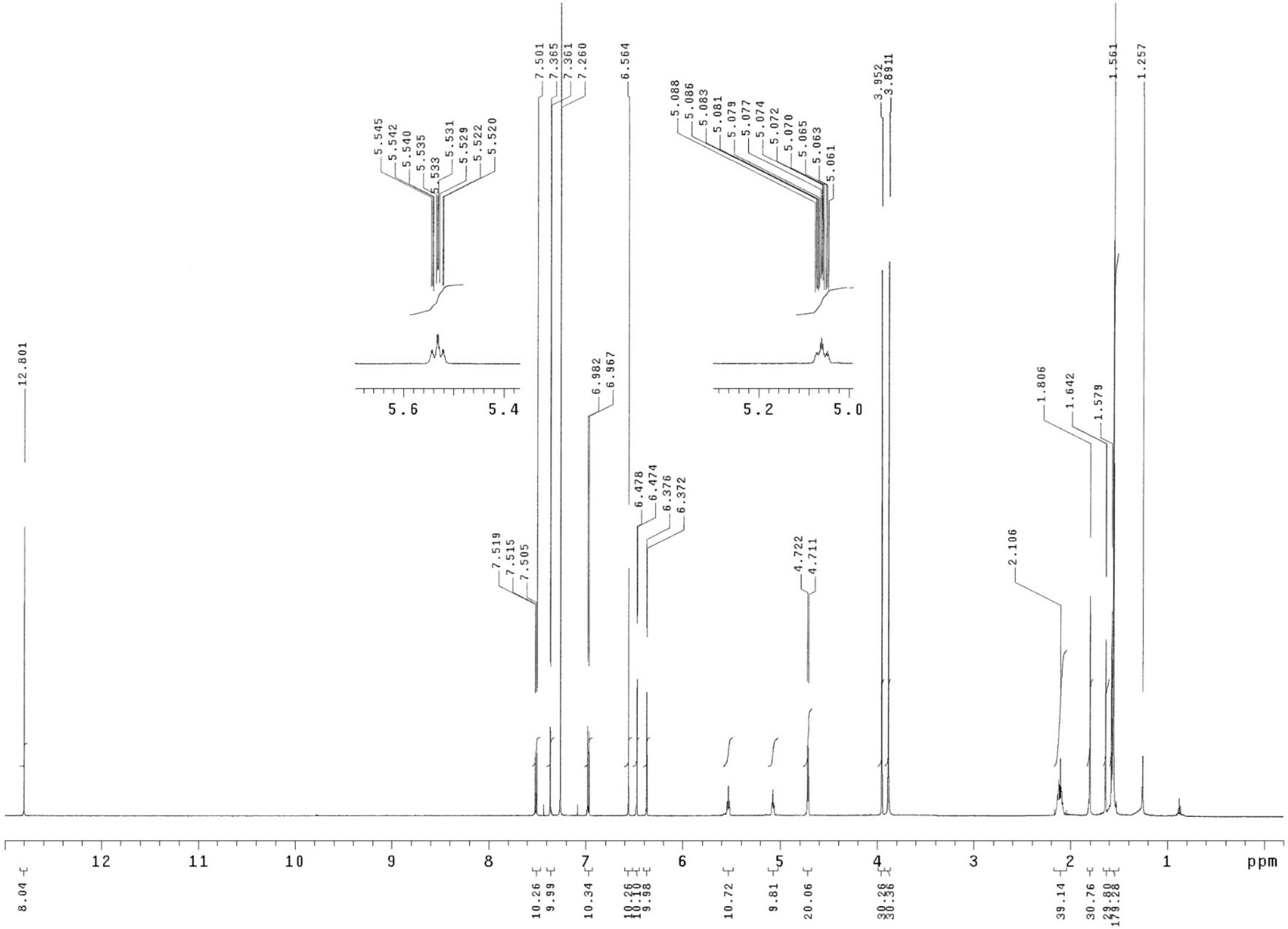

Figure S5. ^{1}H-NMR spectrum (CDCl3, 600 MHz) of **2**.

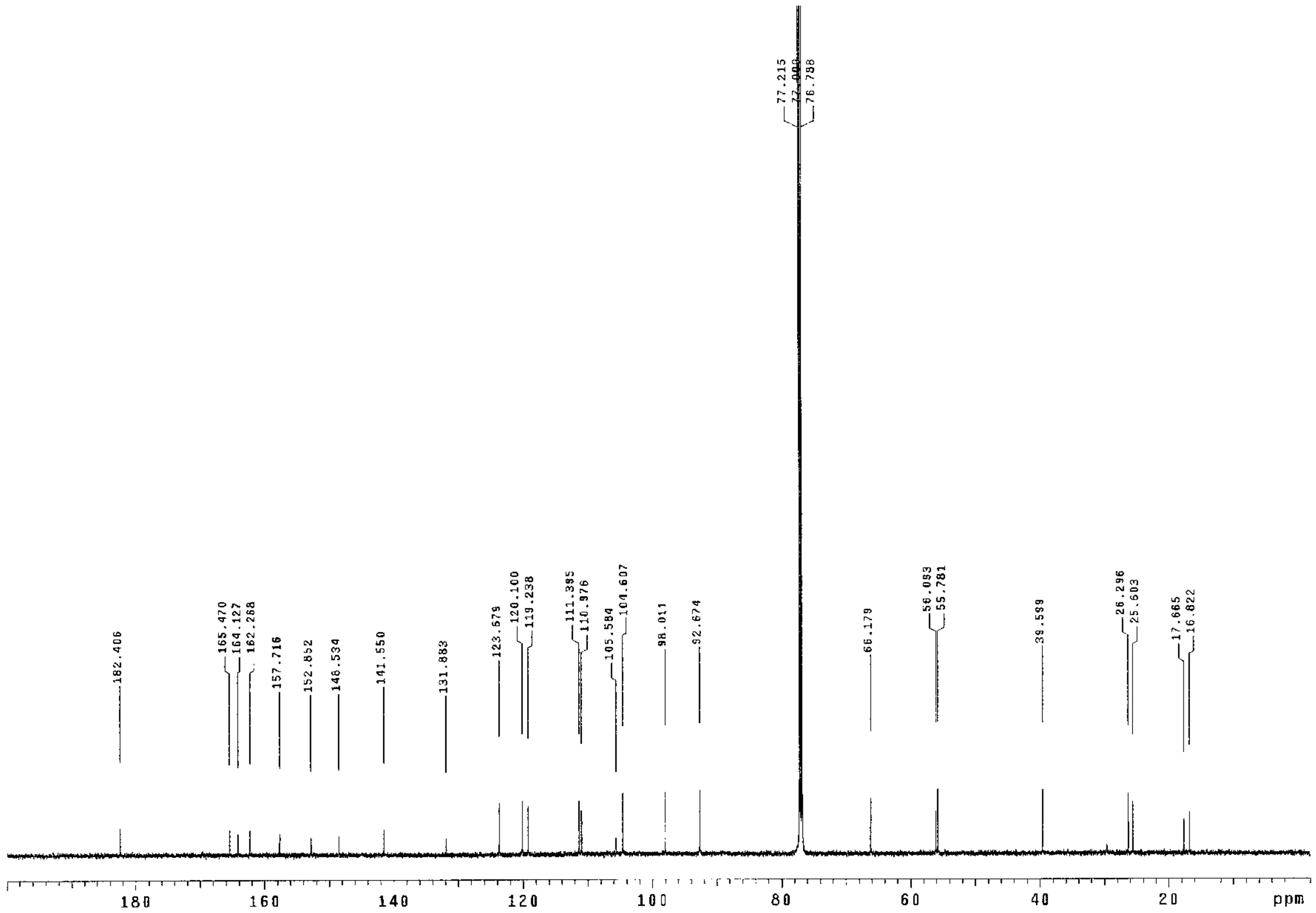

Figure S6. ^{13}C-NMR spectrum ($CDCl_3$, 150 MHz) of **2**.

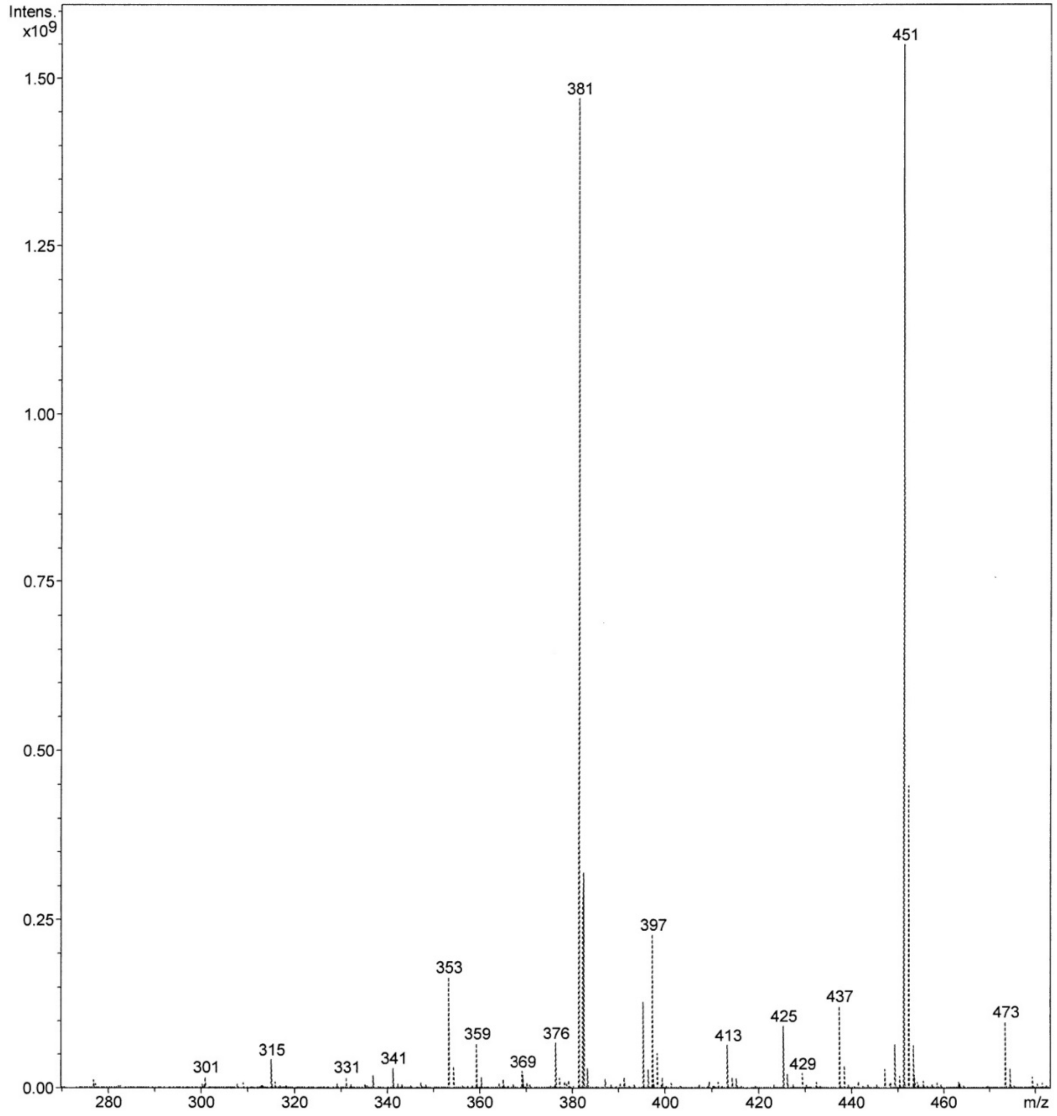

Figure S7. ESI-MS spectrum of **2**.

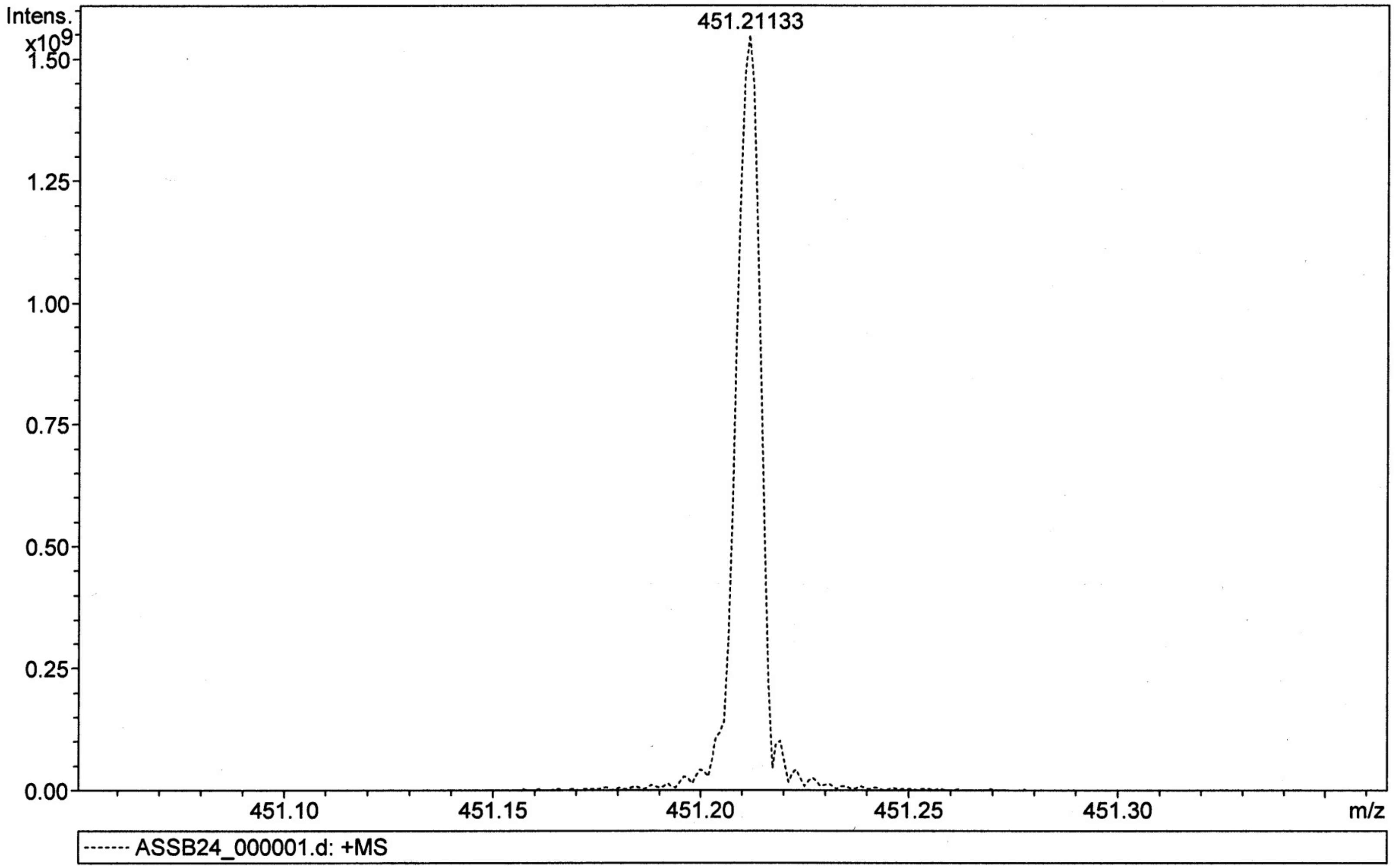

Meas. m/z	#	Formula	Score	m/z	err [mDa]	err [ppm]	mSigma	rdb	e⁻ Conf	N-Rule
451.21133	1	C 27 H 31 O 6	100.00	451.21152	0.18	0.40	7.4	12.5	even	ok

Figure S8. HR-ESI-MS spectrum of **2**.

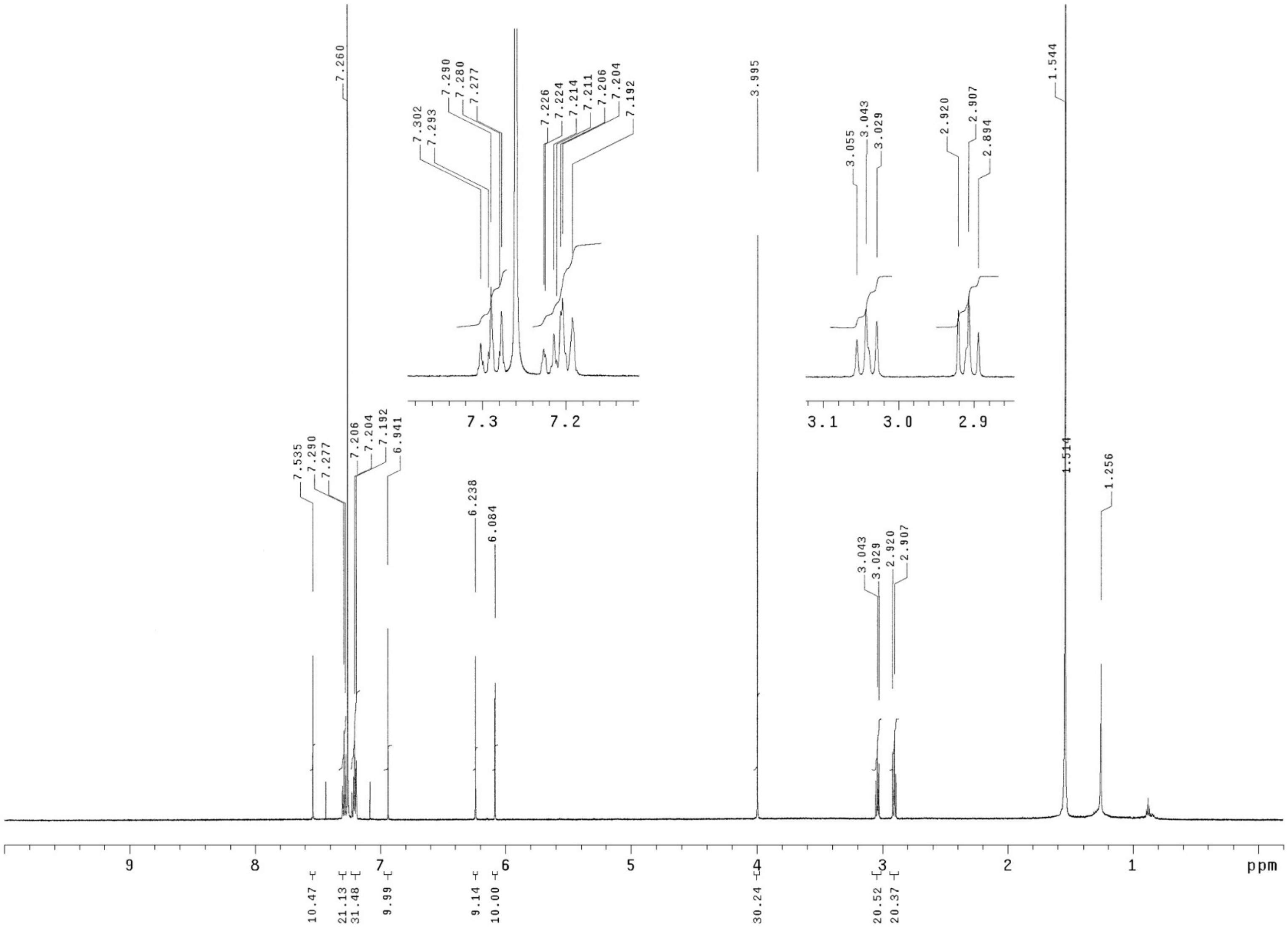

Figure S9. ^{1}H-NMR spectrum ($CDCl_3$, 600 MHz) of **3**.

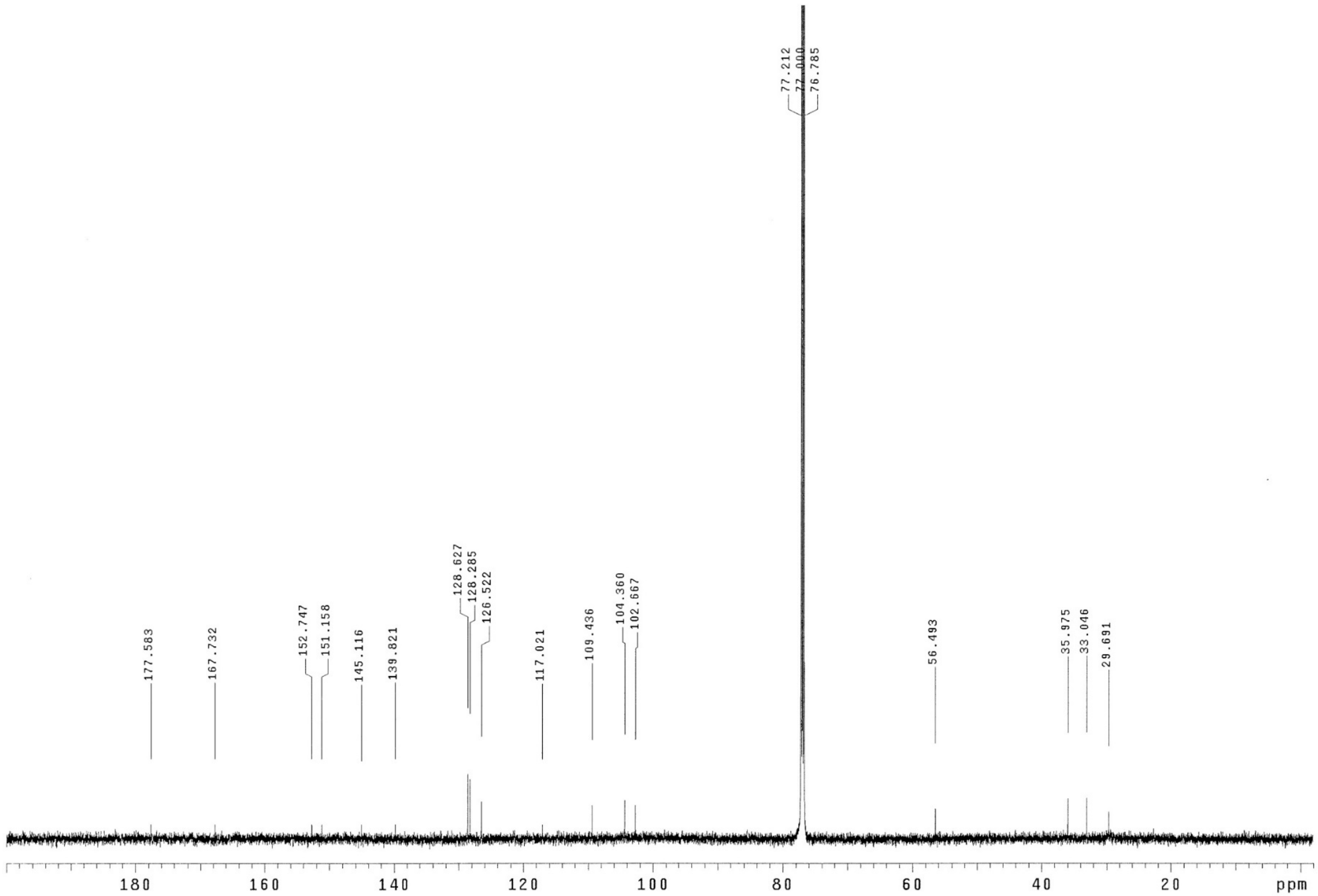

Figure S10. ^{13}C-NMR spectrum of **3** ($CDCl_3$, 150 MHz).

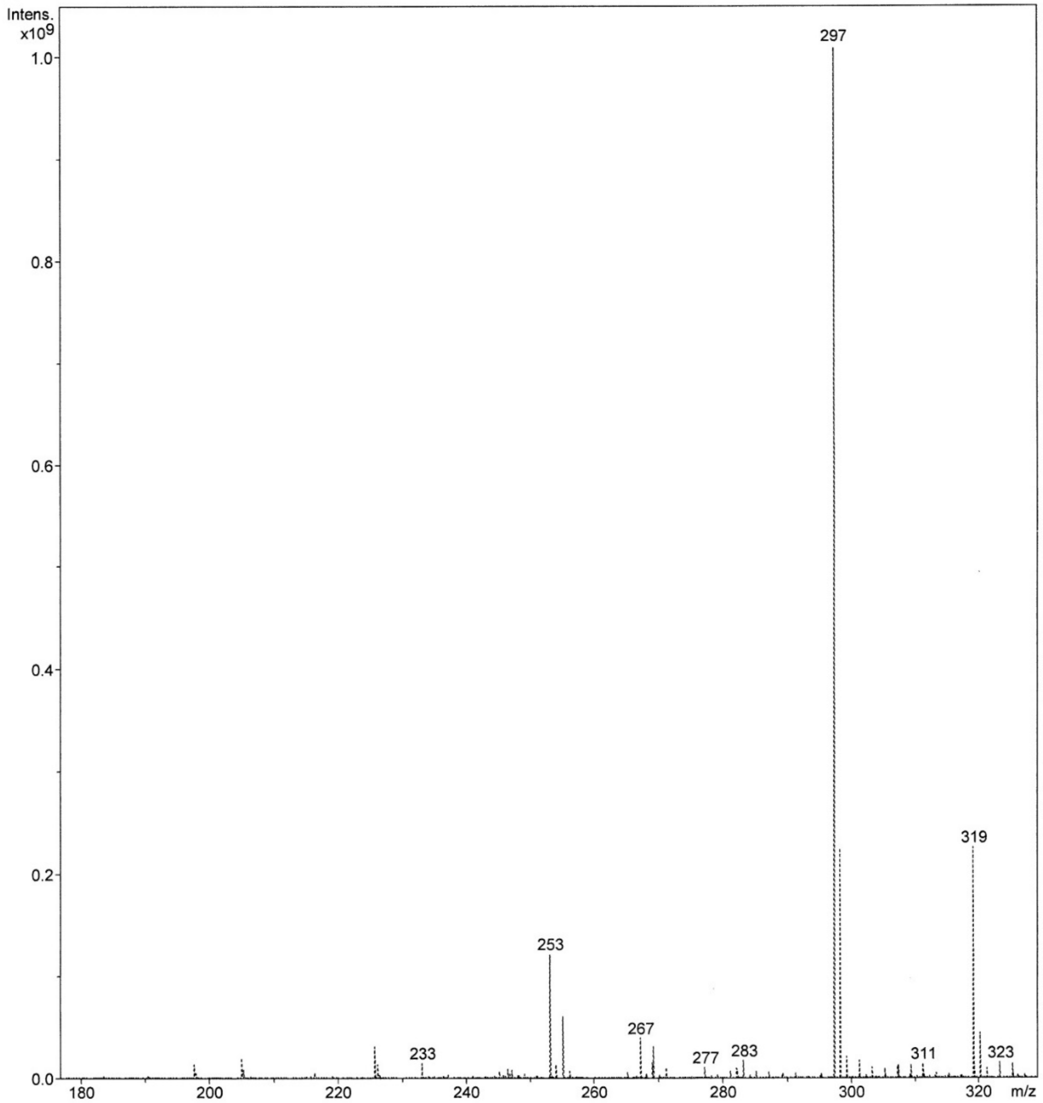

Figure S11. ESI-MS spectrum of **3**.

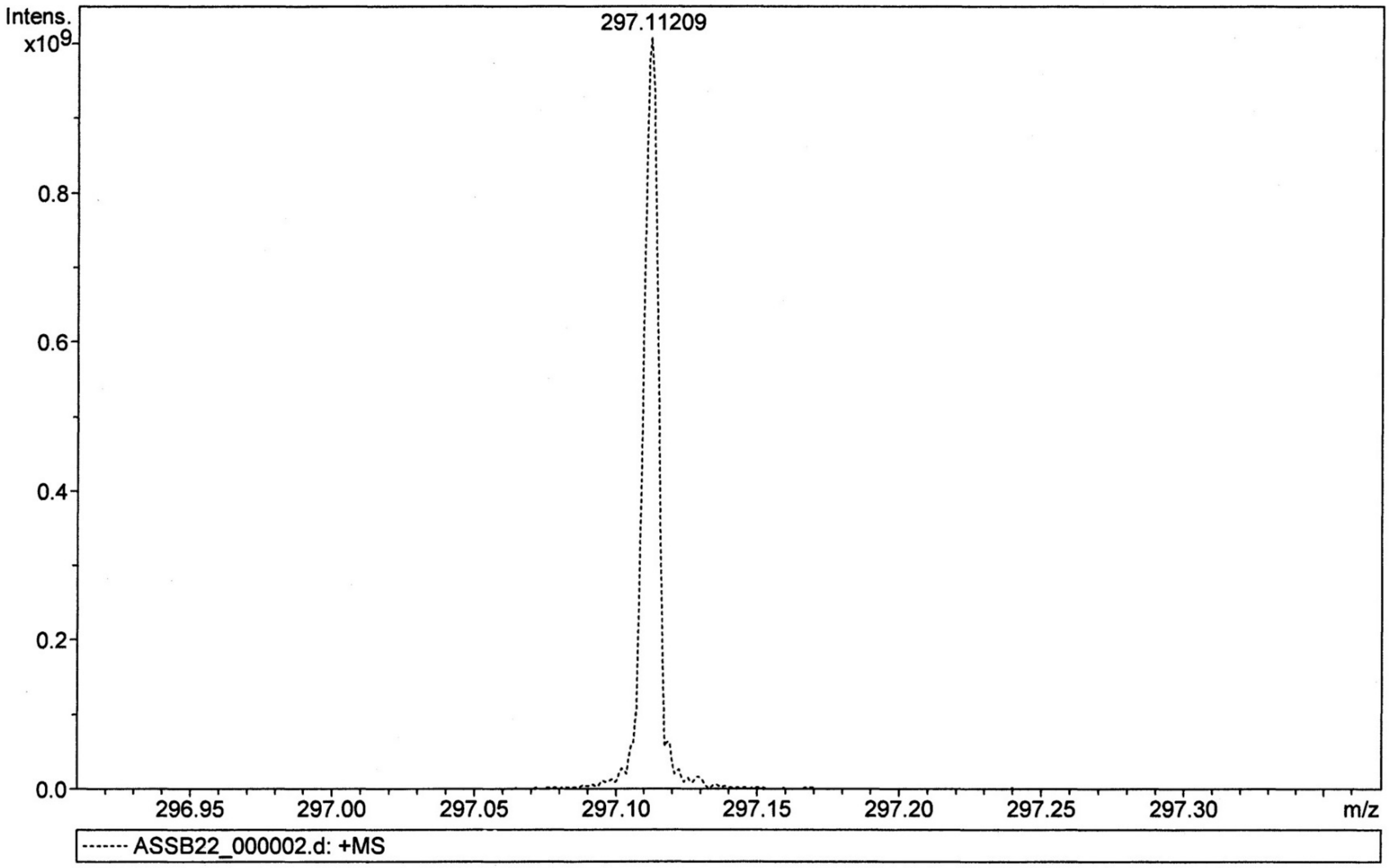

Meas. m/z	#	Formula	Score	m/z	err [mDa]	err [ppm]	mSigma	rdb	e⁻ Conf	N-Rule
297.11209	1	C 18 H 17 O 4	100.00	297.11214	0.04	0.15	16.3	10.5	even	ok

Figure S12. HR-ESI-MS spectrum of **3**.
